# Biological Effects and Toxicity of Compounds Based on Cured Epoxy Resins

**DOI:** 10.3390/polym14224915

**Published:** 2022-11-14

**Authors:** Anna Rudawska, Katarzyna Sarna-Boś, Adrianna Rudawska, Ewa Olewnik-Kruszkowska, Mariaenrica Frigione

**Affiliations:** 1 Faculty of Mechanical Engineering, Lublin University of Technology, Nadbystrzycka 36 St., 20-618 Lublin, Poland; 2 Department of Dental Prosthetics, Medical University of Lublin, Chodźki 6, 20-093 Lublin, Poland; 3 Department of Pharmacology and Toxicology, National Veterinary Research Institute, Aleja Partyzantów 57, 24-100 Puławy, Poland; 4 Faculty of Chemistry, Nicolaus Copernicus University in Toruń, Gagarin 7 St., 87-200 Toruń, Poland; 5 Department of Innovation Engineering, University of Salento, Via Arnesano, 73100 Lecce, Italy

**Keywords:** epoxides, resin-based composites, biological effect, cytotoxic effect, metallic fillers

## Abstract

The aim of this work was to investigate selected biological and toxicity properties of cured epoxy resin-based compounds based on a bisphenol A epoxy resin, cold-cured by a polyamide and containing two types of metal powders (aluminum and copper). This study involved cytotoxicity analysis, pH measurements, absorbance measurements and sterilization. The cytotoxicity analysis was conducted to determine the harmful degree of the cured epoxy resin. Aimed at identifying toxic agents in cured compounds, the cytotoxicity analysis involved absorbance measurements in an entire wavelength range. Cytotoxicity and absorbance results demonstrated that the extracts of all the tested resin samples had no cytotoxic effects on the cells of living organisms. The absorbance values obtained over the entire wavelength range did not point to the formation of aggregations, which proved that no toxic agents harmful to living organisms were extracted from the resin samples. Based on the results obtained, it can be concluded that all tested compounds, based on epoxy resins, which are also used as adhesives in various applications, are essentially safe materials when using such formulations in a cured state.

## 1. Introduction

Epoxy compounds for different technical applications and the methods of their manufacturing were known already in the nineteenth century [1,2,3,4]. Over 85% of the produced epoxy resins are created as a result of the reaction between bisphenol A and epichlorohydrin (ECH) [3,5,6,7]. Launched into production over 50 years ago, epoxy resins aroused enormous interest owing to their valuable properties that distinguished them from other polymeric materials [8,9,10,11]. Due to their unique properties, epoxy resins are applied in many fields of technology; one of the most popular applications is the production of polymer composites that are used as structural elements, including the aviation industry, boatbuilding, construction sector and electric installations [5,6,8,12,13,14,15]. As a result of their polar nature, epoxy resins offer very high adhesion qualities to many different materials, such as glass, ceramics, metal, concrete, and polymers; they are considered, therefore, as universal adhesives [16,17,18,19].

Epoxides are viscous liquids or brittle solids that change their structure during the curing process, and as a result of crosslinking reactions, they become insoluble and infusible, acquiring high strength, good electrical insulating properties and a considerable chemical resistance [3,5,8]. Epoxy resins are the main component of many compounds containing various additives; moreover, with the use of additives or even fillers it is possible, in fact, to produce materials with different features suitable for diverse applications [11,12,13,14,20,21,22,23,24,25,26]. The combination of epoxy resin with fibers yields lighter materials that exhibit metal-like properties, corrosion resistance and durability to ageing [8,12,27,28], or flame retardancy [10,29,30]. In addition, in the medical context, and in particular in dentistry, the interest in epoxy-based materials is due to their ability to better absorb shock and distribute the loads when the resins are filled with other components, which makes them suitable also when used in delicate contexts, such as the immediate loading of newly placed dental implants, as reported by Reda et al. [31].

Due to the numerous applications where epoxy resins can be advantageously exploited, it is necessary to know the properties of such materials and their components, both before and after the curing process. The main characteristics of the resins and of their curing agents, including safety information, are always reported in the data sheets. However, epoxy compounds, once cured, can exhibit slightly different characteristics, in particular, a different toxicity to humans. When producing epoxy-based compounds, on the other hand, the safety of people exposed to the cured materials is a relevant issue; to the best of our knowledge, only limited information is available on this topic.

The toxicology examines the properties of toxic factors and their impact on the body, which are often assessed with biochemical tests. One of the basic tests determines the cytotoxic activity of a given substance. Cytotoxicity measures the ability of a substance to destroy a particular type of cell [32]. The investigation of biological effects on the human body and the toxicity of cured epoxy systems due to their exposure to these materials, may help people who handle these compounds to avoid possible dangers. The analysis of the results obtained from such tests will help to establish effective procedures for the safe handling of such materials. In addition, the chemical and biological properties of various resin-based composites, in relation to the interaction with the human organism, are also important in medical applications [33,34].

The aim of this work is, therefore, to present selected biological and chemical properties of three different cured epoxy compounds, obtaining valuable information for manufacturers and users of such materials.

## 2. Materials and Methods

This work presents a biological, toxicological, and chemical analysis in accordance with the accepted standards of such tests, performed on the appropriate substrates and the appropriate substrates available for biotechnological laboratory tests, which are described in the individual types of tests in this study. No animal or human cells were used in this study.

### 2.1. Epoxy Compounds

The study was performed on samples of three epoxy resin-based compounds, whose composition is summarized in Table 1.

One (unmodified) epoxy compound was based on a bisfenolic epoxy resin (Epidian 53, trade name, producer: CIECH Resins, Nowa Sarzyna, Poland) cured with a polyaminoamide C curing agent (PAC—trade name, producer: CIECH Resins, Nowa Sarzyna, Poland). Two modified epoxy compounds were produced and studied, including a metallic filler (aluminum (Al) or copper (Cu)). The metallic fillers, in powder form, were added in order to improve the resins performance. These compounds were based on the same mixtures of epoxy resins and curing agents as previously described. The amount of filler in each system was selected according to what was carried out in previous experimental works [27,35].

#### 2.1.1. Epoxy Resin

Epidian^®^ 53 epoxy resin is primarily used for saturating, casting, air-tight sealing of electrical equipment and as an impregnating agent in glass fiber laminates and composites; it is also employed as a component for cold-cured structural adhesives. This compound is a mixture of bisphenol A and epichlorohydrin with the addition of styrene, obtaining a mixture possessing an average molecular weight lower than 700 [36]. The mechanical properties of Epidian^®^ 53 epoxy resin are described in [28,36]. The basic physical and chemical properties of Epidian^®^ 53 epoxy resin are listed in Table 2.

Since the subject of this research was to evaluate the biological, toxicological and chemical properties of this resin, Table 2 also lists the toxic components of Epidian^®^ 53 epoxy resin.

Uncured Epidian^®^ 53 resin is reported to be irritating to the skin and eyes, it can cause sensitization when it comes into contact with the skin. This epoxy resin is also dangerous for the natural environment: it is toxic for aquatic organisms and may cause long-term adverse effects in the aquatic environment. The epoxy mixture is flammable and contains styrene, which cany form an explosive mixture with the air. Since styrene vapors are heavier than air, they tend to accumulate in the lower part of rooms, such as basements, canals, and hollows. It should be stored in original, tightly-sealed packaging; in a dry, well-ventilated place away from direct sunlight at a temperature below 25 °C. Under these recommended conditions, this product does not pose any hazard; it does not decompose and it remains stable. Contact with amines and amides, that promote the curing process, should be avoided. Table 3 lists the values of toxicity of this resin.

#### 2.1.2. Polyaminoamide Curing Agent

The polyaminoamide is a polyamide curing agent; its characteristics are given in [35,37]. This curing agent is used for curing liquid, low molecular weight epoxy resins and its compounds. The weight ratio of epoxy resin:curing agent can be adjusted in relation to the properties that need to be achieved in the final cured material: at curing agent content (above 70% wt.) leads to a more elastic and a more impact resistant cured resin, this comes at the expense of its hardness and its resistance to high temperatures (due to the lower glass transition temperature). This curing agent can be employed for cold-cure epoxy resin, i.e., in applications where the curing process is required to take place at room temperature. According to the product’s safety data sheet, this product is caustic, skin irritating and may cause sensitization. It also causes long-term adverse effects in the aquatic environment and it is toxic to aquatic organisms [37].

#### 2.1.3. Powder Metal Fillers: Aluminum (Al) and Copper (Cu)

In some of the epoxy compounds, aluminum or copper metal powder fillers were added in order to improve the resin properties and performance. Electrolytic copper powder may contain particles of lead (max 0.05% wt.), iron (max 0.02% wt.), oxygen (max 0.3% wt.) and residues insoluble in HNO_3_ (max 0.05% wt.). According to the Directive 67/548/EEC and Regulation 1272/2008/EC, it is not classified as harmful or hazardous. However, prolonged exposure to the product in its powdered form may cause irritation to the eyes, skin, and respiratory system. The product is flame retardant. This material must be stored in a dry and well-ventilated room and kept away from humid environments and oxidants. When properly stored and used, it is stable and poses no risk to the user. It cannot be released or spread in the natural environment. Table 4 shows the toxicological information about copper powder.

According to the classification under the Directive 67/548/EEC and Regulation 1272/2008/EC, aluminum powder is highly flammable and releases flammable gases when it comes into contact with water; however, it presents no significant hazards when it comes into contact with skin. Adverse effects are possible: prolonged exposure to dust or fume concentrations will produce irritation to the eyes, skin, and respiratory tract. Dust may cause mechanical irritation to the eyes.

Physical and chemical properties of copper and aluminum powders are listed in Table 5.

### 2.2. Production of Samples for Tests

**Figure 1:**
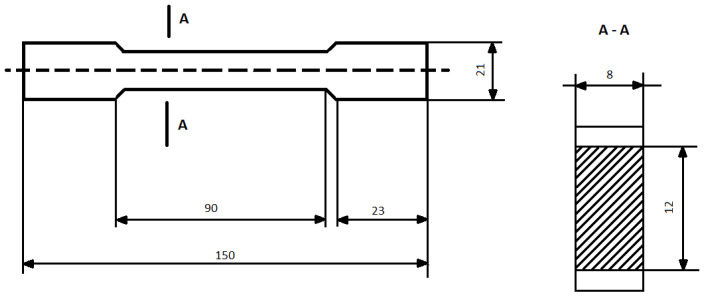
Dimensions (mm) and shape of the specimens of the cured epoxy compounds.

The samples of epoxy compounds were prepared by casting the liquid mixture in a silicone mold with a dog-bone shape. Figure 1 shows the dimensions of the cured epoxy compounds.

The preparation of the epoxy compound samples, with or without each of the fillers, was carried out as follows: (i) casting molds were prepared and cleansed, and then coated with an anti-adhesion agent for polymeric materials, Polsilform^®^ (Polish Silicone, Nowa Sarzyna, Poland); (ii) 100 g of epoxy resin was metered, 2% wt. (2 g) of a metal filler, when used, was added to the resin; (iii) the components were mixed with a high-speed mixer for about 120 s at 460 rev/min; (iv) the compound was left to degas for 120 s; (v) 80 g of polyaminoamide curing agent was then added; (vi) this compound was mixed again for about 120 s and left to degas for an additional 120 s; (vii) the liquid mixture was finally poured into the prepared molds. These epoxy compounds were fabricated at an ambient temperature of 27 ± 3 °C and cured for about 7 days at the same temperature.

### 2.3. Experimental Procedures

#### 2.3.1. Cytotoxicity Analysis

A cytotoxicity analysis was conducted to determine the harmful degree of the cured epoxy resin. This method is used to assess the toxicity levels of a substance, when it is added to a culture medium, in order to determine its activity at the cellular level. It is based on enzymatic reactions that produce a colored product, which is identified spectrophotometrically with the ELISA reader (ELx808™ type—Absorbance Microplate Reader, BioTek, Winooski, Vermont, VT, USA).

The toxicity analysis was performed with the aid of the following laboratory equipment: an incubator with a humid environment at 37 °C and 5% CO_2_, (Binder C150); laminar flow cabinet (biological hazard standard) with HEPA filters, a HEPA H-14 filter capturing 99.999% of particles with a diameter < 0.3 µm and a HEPA filter capturing 99.999% of particles with a diameter > 0.3 µm; water bath at 37 °C; an inverted phase contrast microscope; centrifuge; scales; plate photometer provided with 570 nm filter and shaking mode; Bürker hemocytometer; automatic pipette controller with serological pipettes; automatic pipettes; multi-channel automatic pipettes and vessels for cell cultivation.

The tests were carried out using the following chemical reagents, media and serum: Minimum Essential Medium Eagle ((MEM), without phenol red, glutamine and serum); FBS (serum), penicillin/streptomycin, glutamine; Trypsin-EDTA solution; phosphate buffered saline (PBS); MTT (3-(4,5-Dimethylthiazol-2-yl)-2,5-diphenyltetrazolium bromide); and isopropanol (all analytically pure substances, were provided by Merck KGaA, Darmstadt, Germany).

To ensure proper testing conditions, all solutions, media, vessels for cell cultivation and tools must be sterile. All operations were then carried out in the laminar flow cabinet under sterile conditions. The MTT solution was dissolved in a clean and fresh MEM medium without phenol red at a concentration of 1 mg/mL. The compound was sterilized with the use of a syringe filter and used on the same day. Material samples were extracted from the cured samples of the adhesive compounds. Prior to their use and contact with cells, all fluids were heated in the water bath to 37 °C. On the first day of testing, cells were collected by enzymatic digestion and centrifuged for 3 min. The prepared pellet was suspended in a cell culture medium and diluted to achieve a concentration of 1 × 10^5^ cells/mL. 100 μL of PBS was loaded into the peripheral wells in the 96 well microplate, and 100 μL of the prepared cell suspension was loaded into the other wells. The microplate was incubated for 24 h under the following conditions: 5% CO_2_, 37 °C, humidity > 90%.

On the next day, the microplate was examined under a phase-contrast microscope to verify uniform cell growth. Diluents of the extracts of the tested cured epoxy compounds were prepared, as specified in Table 6. Next, the cell culture media were removed from the microplate, and 100 μL of a relevant solution was added to the wells with the cells, as specified in Table 7.

The microplate was re-incubated for 24 h at 5% CO_2_, 37 °C, humidity > 90%. The next day, the microplate was examined under a phase contrast microscope to verify the cell culture growth.

#### 2.3.2. Absorbance Assay

Aimed at identifying toxic agents in cured compounds, the cytotoxicity analysis involved absorbance measurements in an entire wavelength range. In this experiment, the absorbance assay was performed with the μQuant microplate spectrophotometer (Bio-Tek Instruments, Winooski, Vermont, VT, USA). The µQuant is a single-channel analyzer used for research and development and in vitro diagnostics, it is designed to automatically perform endpoint analysis. The wavelength range is from 200 nm to 999 nm.

50 μL of the MTT solution was added to the wells with cells and incubated at 37 °C for 2 h. After that, the MTT solution was removed from the cells, and 100 μL of isopropanol was added to the wells with the cells. The microplate was incubated at 37 °C for 10 min and then shaken for 1 min; the absorbance was read at a wavelength of 570 nm. Analysis of the results was performed for the OD_570_ of negative control that was greater than 0.2. The difference between the mean OD_570_ of negative control on the left and the right side of the microplate (B2, C2 and D2 versus B11, C11 and D11) should not exceed 15%. Cell viability was calculated based on Equation (1):

viability % = 100 × OD570e/OD570b (1)

where: OD570e—mean OD570 of the samples containing 100% extract; OD570b—mean OD570 of negative controls.

If cell viability drops below 70%, this means that the tested material has cytotoxic properties. The cell viability of the samples containing diluted extracts should be similar or higher than that of the samples containing 100% of the extract. If not, the test should be repeated. The absorbance was measured with 200 μL solutions of each extract (repeated three times) in the wavelength range between 200 nm and 800 nm. Water was used as a reference.

#### 2.3.3. pH Measurement

The pH measurement of the analyzed samples was made with the bench pH meter (type: 1100L, manufactured by VWR pHenomenal^®^, Radnor, PA, USA). The pH meter used in the experiments has an articulated electrode stand and IP 43 rated housing, which provides high resolution and accuracy for precise measurements. It has a large LCD graphic display, with a continuous LED backlight, showing both pH/mV and temperature values simultaneously. The purpose of the pH measurements is to collect information in order to assess the occurrence of risk to humans, as some products are harmful and dangerous at a specific pH.

#### 2.3.4. Sterilization

The main objective of sterilization is to remove all microorganisms on the surface of the materials being studied. An object is considered sterile if the probability of microorganism occurrence is less than 1:1,000,000. The most popular and reliable form of sterilization is steam sterilization: the moist heat acts as a disinfectant, which is able to destroy microorganisms by denaturing their proteins in the cells. Closed in a sealed chamber, saturated vapor reaches a temperature higher than 100 °C, acting as a sterilizing agent. Two variants of the sterilization process were performed in the present study: (i) 121 °C for 15 min; (ii) 134 °C for 3–5 min. The experiments were performed with the use of the BRAVO G4 22L Pro Select, (SCICAN Ltd., Toronto, ON, Canada) autoclave.

## 3. Results

### 3.1. Cytotoxicity Results

The quality control of the test yielded the following results: (i) the mean OD_570_ of the blank >0.2—the criterion is met and is equal to 0.545; (ii) the difference between the mean values of OD_570_ of the blank on the left and right sides of the microplate <15%—the criterion is met and is equal to 1.48%; (iii) cytotoxic positive control—the criterion is met; cell viability equals 45.9%. (For explanation of symbols see Section 2.3.2). Table 8 lists the absorbance values of the tested material extracts, measured with the use of a photometer located on the prepared microplate.

To facilitate the execution of subsequent calculation procedures, the values listed in Table 9 were averaged for the tested epoxy compound samples. They are specified by the sample number and the extract dilution value, i.e.,: “x 1×” indicates the base extract of Sample x; “x 2×” denotes the two-fold diluted extract of Sample x, etc. The cytotoxicity results took into account the sample dilution and maximum possible standard deviations (Table 9). Table 10 gives the cell viability results of the cells treated with extracts of the tested materials obtained from each microplate well. Obtained cytotoxicity results are summarized in given in Table 11.

The test was carried out correctly as indicated by the cytotoxicity assay positive control.

**Figure 2:**
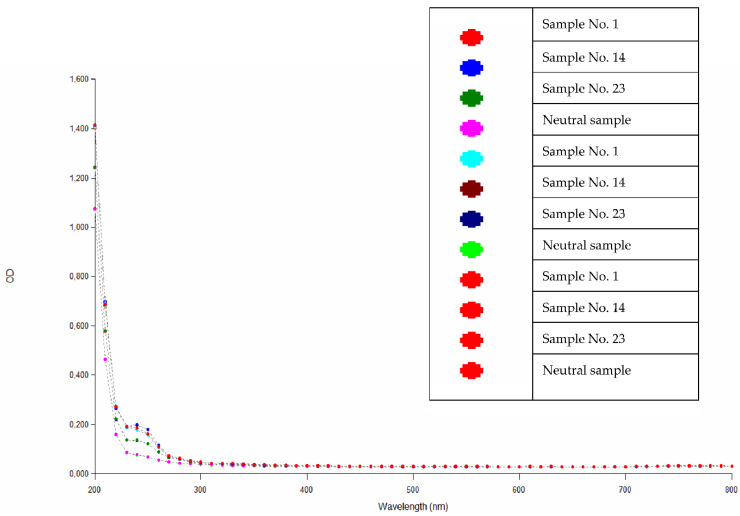
Absorbance values of the extracts of the epoxy compounds.

Absorbance values obtained over the entire wavelength range, shown in Figure 2, prove that there is no extraction of toxic agents from the resin samples, i.e., toxic substances harmful to a living organism and human health.

### 3.2. pH Results

Results of the pH measurements are listed in Table 12.

All samples have acid characteristics. The *p*-value is the probability describing the critical level of significance that corresponds to an acceptable risk of error. Since the significance level used in practice is α = 0.05, this value was also taken in this study. The pH values of Samples 1 and 14 are statistically higher than the benchmark for water because *p* < 0.05. For sample 23 *p* > 0.05, this means that the pH value is statistically insignificant with respect to the benchmark for water.

### 3.3. Sterilization Results

The cured epoxy compounds were subjected to autoclave sterilization at 134 °C for 5 min. The sterilization was aimed at investigating the effect of a specified environment, i.e., an environment with a high temperature, humidity, and pressure, on the structure of the material. Table 13 shows the samples before and after sterilization, in 4-fold magnification.

Neither microscopic examination nor examination with the naked eye showed any changes in the structure, form, or shape of the tested samples of the cured materials. This means that the obtained epoxy compounds are sufficiently resistant and can be used in service conditions, i.e., a typical environment found in medical applications. This test proved, therefore, that even under such harsh conditions, the material does not release any substance. However, this can only be considered a preliminary assessment based on the observation of the photo-micrographs.

## 4. Discussion

Bisphenols (BPs) are produced all over the world in large quantities. They are frequently used in the manufacture of polycarbonate plastics and epoxy resins; such polymers are also used in a variety of consumer products, such as baby feeding bottles, toys, and epoxy liners for food cans [15]. Epoxy resins are also widely used as dental materials, medical adhesive or coatings, bone material and in other medical applications [33,34,39,40,41,42].

At present, most epoxy resins are industrially manufactured from bisphenol A (BPA). Independent studies by vom Saal et al. [43] and Okada et al. [44] have shown that BPA is a hormonally active agent: it exhibits estrogen-like behavior and causes human endocrine disorders, such as precocious puberty. BPA has been acknowledged to be an estrogenic chemical that is able to interact with human estrogen receptors (ER). This is particularly important because ERR-γ is expressed very strongly in the mammalian fetal brain and placenta at sites that could have important outcomes for newborns. Ikhlas et al. [45] also found that bisphenols are capable of inducing cytotoxicity through oxidative stress and genotoxicity. Therefore, the harmful effects of BPA on human health and the environment force researchers to find a BPA substitute. O’Boyle et al. [32] carried out similar investigations on bisphenol A (DGEBA) and bisphenol F (DGEBF), which is widely used as components in epoxy resin thermosetting products, with regard to contact allergy and cytotoxicity. They are known to cause both occupational and no occupational allergic contact dermatitis. It was found that the allergenic effects of DGEBF were dependent on its terminal epoxide groups. It was also shown that the cytotoxicity in a monolayer cell culture was dependent, not only on the presence of epoxide groups, but also on other structural features. Lee et al. [22] evaluated the physical properties and cytotoxicity of a novel root-end filling material (EPC), which was made from epoxy resin and Portland cement, as a mineral trioxide aggregate (MTA) substitute. On the basis of the results on cell viability and morphological changes, it can be noted that this new EPC cement has low cytotoxicity and favors capable biocompatibility. Based on physical and biological results, a root-end filling material (EPC), would be useful in clinical applications. On the other hand, Kostoryz, et al. [46] investigated the effect of adding spiroorthocarbonate (SOC) or polyol on the cytotoxicity of epoxy-based dental resins and found that the addition of SOC and polyols to epoxy-based resin formulations can contribute to the development of biocompatible dental composites. SOC and polyols reduced the cytotoxicity of some epoxy-based dental resins, suggesting that improved polymerization may have taken place in these epoxy-based formulations.

A complete knowledge of the structural features that contribute to the allergenic and cytotoxic effects of DGEBF will guide the development of future novel epoxy resin systems with reduced health hazards when in contact with them. Cytotoxicity and absorbance analysis, of the three cured epoxy compounds, performed in this study demonstrated that the extracts of all the tested resin samples had no cytotoxic effect on the cells of living organisms. Correctness of the test was verified by the cytotoxicity assay positive control. The absorbance values obtained over the entire wavelength range did not point to the formation of aggregations, which proved that no toxic agents harmful to living organisms were extracted from the resin samples. The pH measurements revealed that all tested samples were acidic. Both the microscopic and naked-eye examinations of the autoclaved sterilized samples showed no changes in the structure, form, or shape of the tested cured resin samples.

## 5. Conclusions

Cytotoxicity and absorbance results demonstrate that the extracts of all the tested resin samples have no cytotoxic effect on the cells of living organisms. Correctness of the test was verified by the cytotoxicity assay positive control. The absorbance values obtained over the entire wavelength range do not point to the formation of aggregations, which proves that no toxic agents harmful to living organisms are extracted from the resin samples. Both the microscopic and naked-eye examinations of autoclave sterilized samples showed no changes in the structure, form, or shape of the tested cured resin compound samples. Summing up, it can be concluded that:

- Results of this experimental study demonstrate that cured epoxy resins are safe materials, even in contact with human cells;
- Cured epoxy compounds are homogeneous, monolithic structures. The material is coherent and exhibits typical mechanical strength properties;
- Additives such as metal powders do not cause deviations in compound curing.

## Figures and Tables

**Table 1:** Compositions of epoxy compounds.

Epoxy Resin Content (g)	Curing Agent Content (g)	Type of Powder Filler	Filler Content (g)	Denotation of Resin-Based Compound
100	80	-	-	E53/PAC/100:80
100	80	aluminum	2	E53/PAC/Al/100:80:2
100	80	copper	2	E53/PAC/Cu/100:80:2

**Table 2:** Physical and chemical properties and toxic components of Epidian 53 [37].

Property			Characteristic/Value		
Form			Pale yellow, highly viscous liquid		
Odor			Weakly perceptible		
pH value			7 pH		
Density at 20 °C			1.11–1.15 g/cm^3^		
Viscosity at 25 °C			900–1500 mPa		
Water solubility			Insoluble		
Toxic components of Epidian^®^ 53 epoxy resin					
Compounds	CAS number	WE number	Symbol ^1^	R-phrases ^1^	% wt.
Epoxy resin (average molecular weight ≤ 700)	25068-38-6	500-033-5	Xi, N	36/38-43-51/53	>50%
Styrene	100-42-5	202-851-5	Xn, Xi	10-20-36/38	<12.5%

^1^ Legend: Xi—irritant, Xn—harmful, N—dangerous for the environment, R 36/38—irritating to eyes and skin, R 43—may cause sensitization in skin contact, R 51/53—toxic to aquatic organisms.

**Table 3:** Toxic properties of Epidian^®^ 53 epoxy resin [37].

Property	Compound	Characteristic/Value
Inhalation	Epoxy resin LC_50_	No data available
	Styrene LC_50_ (rat) 4 h	24,000 mg/m^3^
	Styrene TCL_0_ (human)	2600 mg/m^3^
	Styrene LCL_0_ (human)	43,000 mg/m^3^
Skin	Epoxy resin LD_50_ (rat)	>2000 mg/kg
	Styrene LD_50_	No data available
Ingestion	Epoxy resin LD_50_ (rat)	>2000 mg/kg
	Styrene LD_50_ (rat)	5000 mg/kg

**Table 4:** Information from safety data sheet of copper powder [38].

Property	Description	Value/Characteristic
Derived non-effect levels (DNEL)	long-term, oral, dermal	0.041 mg/kg body weight/day
	acute, oral, dermal	0.082 mg/kg body weight/day
Predicted non-effect concentrations (PNEC)	sediment—freshwater	87 mg/kg dry matter
	sediment—marine water	676 mg/kg dry matter
	soil	65.5 mg/kg dry matter

**Table 5:** Physical and chemical properties of copper and aluminum powders [38].

Property	Characteristic/Value	
	Type of Powder	
	Copper	Aluminum
Physical state/Appearance	Solid/Powder	Solid/Powder
Color	Copper	Silver
Odor	Odorless	Odorless
Melting point/Freezing point	1083 °C	660 °C
Flammability (solid, gas)	Non-flammable	Flammable
Upper/Lower flammability or explosive limits	No data available	No data available
Water solubility	Insoluble	Water insoluble, in contact with water releases flammable gas; insoluble in organic solvents
Explosive properties	Risk of dust explosion	Risk of dust explosion
Bulk density	1.2–3.5 g/cm^3^	-
Particle size	0.063 mm	0.063 mm

**Table 6:** Extract concentrations of cured epoxy compounds.

Concentration	Extract Volume	MH ^1^ Volume
1×	500 µL	0 µL
2×	250 µL	250 µL
3×	170 µL	330 µL
4×	125 µL	375 µL

^1^ MH—cell culture medium.

**Table 7:** Schematic design of a 96 well microplate used for examination of extracts of the three types of cured epoxy compounds.

No.	1	2	3	4	5	6	7	8	9	10	11	12
A	PBS	PBS	PBS	PBS	PBS	PBS	PBS	PBS	PBS	PBS	PBS	PBS
B	PBS	B	A1×	A1×	A1×	A1×	A2×	A2×	A2×	A2×	B	PBS
C	PBS	B	A3×	A3×	A3×	A3×	A4×	A4×	A4×	A4×	B	PBS
D	PBS	B	B1×	B1×	B1×	B1×	B2×	B2×	B2×	B2×	B	PBS
E	PBS	K−	B3×	B3×	B3×	B3×	B4×	B4×	B4×	B4×	K+	PBS
F	PBS	K−	C1×	C1×	C1×	C1×	C2×	C2×	C2×	C2×	K+	PBS
G	PBS	K−	C3×	C3×	C3×	C3×	C4×	C4×	C4×	C4×	K+	PBS
H	PBS	PBS	PBS	PBS	PBS	PBS	PBS	PBS	PBS	PBS	PBS	PBS

Legend: K−—negative control, K+—positive control, A1×—extract of medical product A, undiluted, A2×—extract of medical product A, 2-fold diluted, A3×—extract of medical product A, 3-fold diluted, A4×—extract of medical product A, 4-fold diluted, B1×—extract of medical product B, undiluted, B2×—extract of medical product B, 2-fold diluted, B3×—extract of medical product B, 3-fold diluted, B4×—extract of medical product B, 4-fold diluted, C1×—extract of medical product C, undiluted, C2×—extract of medical product C, 2-fold diluted, C3×—extract of medical product C, 3-fold diluted, C4×—extract of medical product C, 4-fold diluted, B—blank (bottled medium cells).

**Table 8:** Absorbance values of microplate wells in extract analysis.

No.	1	2	3	4	5	6	7	8	9	10	11	12
A	0.266	0.264	0.259	0.262	0.251	0.266	0.262	0.263	0.262	0.261	0.265	0.271
B	0.252	0.516	0.499	0.532	0.594	0.536	0.568	0.576	0.573	0.543	0.535	0.260
C	0.248	0.535	0.556	0.598	0.644	0.625	0.599	0.607	0.588	0.576	0.546	0.266
D	0.258	0.572	0.594	0.606	0.644	0.632	0.632	0.623	0.612	0.573	0.566	0.268
E	0.253	0.550	0.590	0.616	0.665	0.646	0.631	0.641	0.614	0.590	0.248	0.269
F	0.259	0.555	0.583	0.611	0.621	0.637	0.635	0.635	0.611	0.583	0.249	0.271
G	0.263	0.538	0.566	0.577	0.669	0.612	0.582	0.576	0.586	0.560	0.254	0.283
H	0.278	0.275	0.276	0.275	0.269	0.279	0.279	0.282	0.279	0.279	0.280	0.284

**Table 9:** Absorbance values of microplate wells in extract analysis.

Sample											
1 1×	1 2×	1 3×	1 4×	14 1×	14 2×	14 3×	14 4×	23 1×	23 2×	23 3×	23 4×
Cytotoxicity											
99.1	103.7	111.1	108.7	113.6	111.9	115.5	113.6	112.5	113.0	111.2	105.7
Standard Deviation											
7.2	2.8	7.0	2.5	4.2	4.8	6.1	4.1	4.2	4.5	8.5	2.1
Positive control											
45.9 (0.6)											

**Table 10:** Cell viability results.

No.	1	2	3	4	5	6	7	8	9	10	11	12
A	91.6	97.6	109.0	98.3	104.2	105.7	105.1	99.6	105.3	97.9	107.1	105.5
B	102.0	109.7	118.2	114.7	109.9	111.4	107.9	105.7	108.2	114.2	107.7	106.3
C	109.0	111.2	118.2	116.0	116.0	114.3	112.3	105.1	109.7	112.4	114.8	104.7
D	108.3	113.0	122.0	118.5	115.8	117.6	112.7	108.3	109.1	110.7	114.5	116.3
E	107.0	112.1	113.9	116.9	116.5	116.5	112.1	107.0	110.6	112.2	114.6	111.1
F	103.9	105.9	122.8	112.3	106.8	105.7	107.5	102.8	105.5	106.1	111.7	104.8
G	106.7	107.3	113.6	111.4	103.8	107.2	110.3	103.5	107.7	107.2	112.4	105.2
H	104.5	109.4	114.0	109.8	104.7	105.3	107.1	106.6	104.9	106.9	113.3	104.8

**Table 11:** Summary of cytotoxicity results.

Material	Cell Viability	Criteria
Positive control	45.9	Satisfied
Negative control	100.5	Satisfied
Samples: 1 1×–23 4× (Table 11)	99.1–105.7	No Cytotoxicity
OD_570_	0.545	Satisfied
Differences in cell growth	1.48%	Satisfied

**Table 12:** pH values.

Samples	Test I	Test II	Test III	Mean	Standard Deviation	pH Value	*p*
Sample no. 1	6.115	6.104	6.086	6.102	0.015	6	0.039
Sample no. 14	6.087	6.015	6.077	6.060	0.039	6	0.045
Sample no. 23	5.750	5.896	5.942	5.863	0.100	6	0.733
Water	5.803	5.910	5.956	5.890	0.079	6	1.000

**Table 13:** Unmodified and filler modified epoxy compounds after curing and after sterilization.

Epoxy Compound	Condition	
	After Curing	After Sterilization
Epidian53/PAC/100:80—unmodified	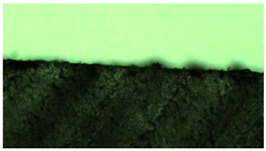	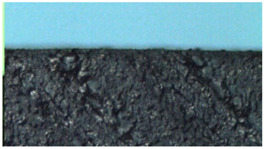
Epidian53/PAC/Al/100:80:2—modified	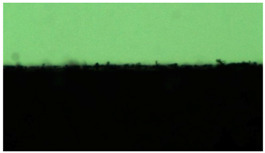	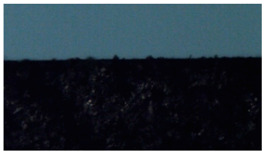
Epidian53/PAC/Cu/100:80:2—modified	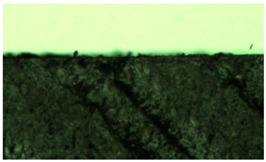	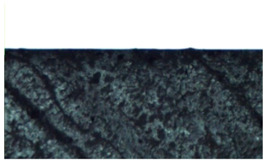

## Data Availability

Not applicable.
